# Study on the Localization of Fangcang Shelter Hospitals During Pandemic Outbreaks

**DOI:** 10.3389/fpubh.2022.876558

**Published:** 2022-06-21

**Authors:** Bin Hu, Wei Chen, Tingyu Yue, Guanhua Jiang

**Affiliations:** ^1^School of Economics and Management, China University of Mining and Technology, Xuzhou, China; ^2^School of Public Health, Xuzhou Medical University, Xuzhou, China

**Keywords:** pandemic outbreak, COVID-19, healthcare management, Fangcang shelter hospital, EWM-TOPSIS method, hierarchical progressive location, two-level hierarchical model

## Abstract

In the event of pandemic, it is essential for government authority to implement responses to control the pandemic and protect people's health with rapidity and efficicency. In this study, we first develop an evaluation framework consisting of the entropy weight method (EWM) and the technique for order preference by similarity to ideal solution (TOPSIS) to identify the preliminary selection of Fangcang shelter hospitals; next, we consider the timeliness of isolation and treatment of patients with different degrees of severity of the infectious disease, with the referral to and triage in Fangcang shelter hospitals characterized and two optimization models developed. The computational results of Model 1 and Model 2 are compared and analyzed. A case study in Xuzhou, Jiangsu Province, China, is used to demonstrate the real-life applicability of the proposed models. The two-stage localization method gives decision-makers more options in case of emergencies and can effectively designate the location. This article may give recommendations of and new insights into parameter settings in isolation hospital for governments and public health managers.

## Introduction

Pandemic outbreaks have posed a threat to humanity and economic progress (1). In 2020, the coronavirus disease 2019 (COVID-19) pandemic broke out. By February 15, 2022, ~409.21 million cumulative cases have been diagnosed worldwide, with an approximate of 71.35 million existent confirmed cases and 5.81 million cumulative deaths. COVID-19 has led to substantial loss and threat to economies (2) and people's lives and health (3).

As of the onset of the COVID-19 pandemic worldwide, conventional methods of response have been invalid and inffeicient, as countries are generally encountered with uneven distribution of healthcare resources, operational inefficiencies, lack of flexibility, and shortages of hospital beds (4). To minize the transmission of COVID-19, the World Health Organization and the majority of governments have recommended “stay-at-home” (5). Due to the absence of a prophylactic vaccine, official control measures have been implemented to reduce the spread of COVID-19, such as staying at home (6). People who may have been exposed to COVID-19 are instructed to subject themselves to household quarantine for at least 14 days, which effectively prevents them from close contact with others as well as from going to school and workplace or any public venue. Arguably, self-isolation could be effective in retarding the spread of this contagious disease, as was the case with SARS in 2003 (7). China has implemented some drastic control measures that substantially mitigated the spread of the disease (8). Western mitigation interventions have been less effective and multiple measures of social distancing and self-isolation may be required to adequately control COVID-19 (9). Self-isolation at home has been demonstrably one of the most effective public health interventions and measures (10). Dickens et al. (11) described the effectiveness of isolation measures, which simulated and compared two kinds of isolation profiles: institution-based isolation and home-based isolation, both can reduce the spread of infectious diseases and decrease mortality, with institutional isolation providing better containment of COVID-19.

Meanwhile, home-based isolation may pose two problems. (i) Homebound patients can lead to the spread of infectious diseases in the community. Homebound patients will be in close contact with their families, with their family members being a potential source of the pandemic spread. Moreover, patients restricted to home-based isolation may still move around and contact others in the community, ultimatel\ leading to the spread of infectious disease in the community and further increased number of infectious cases (12). (ii) Isolation of patients at home may delay the best time for life-saving of infectious disease, with patients susceptible to deterioration from mild-to-moderate or severe disease (13). Patients under home-based isolation do not have access to monitoring of appropriate symptom and necessary and timely hospital referrals. Without access to appropriate medical care, patients with rapidly progressing diseases will further burden the healthcare system. These factors will continue to lead to a shortage of medical resources and beds, resulting in many cases exceeding the local medical capacity, as is currently the case in several parts of the world. Therefore, pandemic outbreak challenges response and control capacity, which must include timely isolation of infected individuals and timely admission of various types of patients to preclude new sources of infection and protect people's health (14).

The successful renovation of Fangcang shelter hospitals in Wuhan has demonstrated the unique role of Fangcang shelter hospitals in responding to a pandemic outbreak, especially in the case of the pandemic widespread. When there are many patients with mild-to-moderate disease, Fangcang shelter hospitals can effectively cope with the problems of isolation of infectious patients and solution to hospital bed shortages. This is the most effective means to control the virus spread and reduce mortality. Such shelter hospitals will rapidly improve the function of the community health care system and improve local medical capacity during a pandemic, providing a good schema for future pandemic prevention and other regional responses to public health emergencies.

Now, COVID-19 prevalence has been worldwide, particularly in areas such as New Delhi and Mumbai in India. However, local governments did not establish medical facilities like Fangcang shelter hospitals promptly and effectively, which was responsible for the increasing cases of COVID-19 infection and the death toll. By February 15, 2022, there were ~0.42 million existent cases of COVID-19 in India, with a cumulative approximate of 42.69 million cases and around 0.5 million deaths. Had timely measures been taken earlier in the outbreak, such as the establishment of Fangcang shelter hospitals, the current serious situation could have been prevented or at least mitigated. However, the localization of shelter hospitals is not arbitrary. On the one hand, unlike conventional hospitals, Fangcang shelter hospitals are unique in location requirements on the grounds of exclusive admission and treatment of patients with infectious diseases. In decision-making of location option, some relevant factors are prerequisite. Moreover, the localization and establishment of Fangcang shelter hospital must ensure the rapid isolation of infected individuals, prevention from infection, provision of healthcare and protection of the population at large. On the other hand, Fangcang shelter hospitals have admission criteria distinct from designated higher-level hospitals with respect to severity of infectious diseases, with Fangcang shelter hospitals targeting acute and mild-to-moderate cases. The questions in research are thus proposed.

**Question 1:** How do we develop the evaluation framework of the preliminary selection of Fangcang shelter hospitals according to the actual situation and requirements of pandemic control?
**Question 2:** How do we determine the final optimal location of Fangcang shelter hospitals to ensure that infectious patients with different severities are quickly and timely allocated to appropriate hospitals early in the outbreak?

Due to the limitation of data sources and operational feasibility, we designated Xuzhou to exemplify and verify the validity of the two-stage location method in this study and provide recommendation and reference for local authorities as well as the institutions abroad.

## Literature Review

More and more attention is being paid to healthcare management to make informed decisions (15). This study is closely related to the literature on the location of field hospitals and temporary medical centers. These hospitals are usually used for support after disasters and epidemics (16). Salman et al. adopted a multi-period mixed integer programming model to optimize capacity allocation and casualty transportation decisions. The model was intended to minimize the total time for travel and waiting of casualties during search and rescue and the total cost of establishing new field hospitals (17). Aydin et al. developed a two-stage stochastic *p*-median model to identify the number, location, and size of field hospitals and the distribution of victims. The objective function minimizes the expected total travel distance of victims (18). Fereiduni et al. addressed the location problem of temporary medical centers and hospitals, considering both distribution and evacuation issues. The objective was to minimize total transportation costs, inventory costs, and facility setup costs (19). Oksuz et al. aimed to identify the location and number of temporary medical centers. They considered the locations of existing hospitals, patient capacities of existing hospitals and temporary medical centers, the setup costs of temporary medical centers, the costs of casualty transportation, and the expected numbers of casualties in the affected areas. The objective was to minimize the total transportation costs and total setup costs of medical centers (20).

Healthcare management in the context of past epidemic or pandemic outbreaks has received considerable scholarly attention. Scholars have addressed many problems to ensure the safety and health of people's lives. Lee et al. modeled the location of point-of-dispensing facilities within cities during an anthrax outbreak for the orderly, rapid, and safe distribution of prophylactic drugs to populations. The proposed model aimed to minimize the average travel distance for all households (21). Araz et al. considered the location of Point of Dispensing sites and the allocation of staff to the selected locations. They formulated a *p*-median facility location model with a queuing approximation to minimize individuals' average transportation and waiting duration to receive the required service (22). Büyüktahtakin et al. developed an epidemiological mixed integer planning model, considering the spatial transmission dynamics of infectious diseases. They identified the location of Ebola treatment centers and optimized logistics for controlling infectious disease outbreaks. The model objective was to minimize the total number of infections and deaths over multiple planning periods with a limited budget (23). Liu et al. modified Büyüktahtakin's model (23) by changing the capacity constraints and applied it to control the 2009 H1N1 pandemic in China. They developed a mixed integer non-linear programming model to determine when to open isolation wards and close unused isolation wards (1). Anparasan et al. modeled the location of cholera treatment facilities based on the 2010 cholera outbreak in Haiti. The objective was to maximize the movement of severely diseased patients from the triage node to treatment facilities, reducing morbidity and mortality and decreasing the total number of deaths from cholera (24).

COVID-19 is a typical pandemic, with arising studies focused on the facility location in the context of COVID-19 (25). Devi et al. proposed a mixed integer linear programming model to identify the location of temporary testing laboratories for COVID-19. The first objective rests on minimizing the total cost, whereas the second objective minimizes the maximum travel time between a temporary testing laboratory and a demand node (26). Risanger et al. cited the distance-determined willingness to travel function of residents and set pharmacies as testing facilities. They developed a model to maximize the number of people going to the nearest selected pharmacy (27). Liu et al. proposed a two-phase framework consisting of pre-and post-pandemic decisions to locate testing facilities and adjust capacity during large-scale emergencies. The framework can meet the dynamic and varying demands caused by pandemics (28). Çakir et al. addressed the allocation problem of mobile vaccination clinics set up in larger cities in Turkey. They employed a spherical bipolar fuzzy multiple-criteria decision-making (MCDM) method to calculate weight and minimized demand allocation and facility setup costs (29). Bertsimas et al. addressed the vaccination facility location problem with a new data-driven approach, where they first improved the DELPHI epidemiological model to capture the impact of vaccination and the variation of mortality by stratified age groups. The predictive model was then integrated into a location-allocation model to optimize the location of vaccination facilities and vaccine distribution. The proposed model aims to minimize the number of deaths, the number of susceptible contacts, and the distance to a facility (30).

There is arising literature on the location of hospitals in the context of COVID-19: Hashemkhani et al. identified the locations of isolation hospitals during the COVID-19 outbreak through a gray-based decision support framework (31). Aydin et al. proposed a DELPHI-based multicriteria decision-making (MCDM) framework, an integrated framework consisting of the DELPHI, Best Worst, and interval type-2 fuzzy logic TOPSIS methods. Better and safer infectious disease services for patients with mild-to-moderate symptoms can be provided by screening the best locations for isolation hospitals (32). Akpinar et al. (33) applied the fuzzy Choquet integral multicriteria decision-making technique to determine the most suitable location for a COVID-19 field hospital to be constructed in Izmir, Turkey. Hassan et al. (34) proposed a variant of the maximal coverage location model to establish field hospitals for the COVID-19 problem, and the objective was to maximize the number of patients covered by the field hospitals.

The main functions of Fangcang shelter hospital include isolation, triage, basic medical services, symptom monitoring, rapid referral, and accommodation and shelter for patients with infectious diseases (35). In Wuhan, China, there were many patients with mild-to-moderate diseases, which led to a shortage of hospital beds at the onset of the pandemic. Accordingly, more than 20 Fangcang shelter hospitals were built to increase capacity. Fang et al. described the success of Wuhan in combating COVID-19 and indicated that Fangcang shelter hospitals, which are usually converted from large public places and monolithic buildings, such as gymnasiums and exhibition centers, are an effective solution to the spread of infectious disease and mortality reduction. Moreover, by establishing Fangcang shelter hospitals, the medical treatment capacity can be increased with less cost (4).

Despite these advantages, there are several challenges with the use of Fangcang shelter hospitals. First, some individuals were unaccustomed to the activity restriction and the reduced interpersonal interactions (36). Also, the physical inactivity caused by isolation interventions has some negative effects (37). Consequently, they were reluctant to be isolated in Fangcang shelter hospital. Second, there is an increased level of worry and nervousness among the isolated people (38). There are emotional and psychological problems associated with these periods of quarantine (39). Facility-based isolation leaves negative psychological effects on people, including post-traumatic stress symptoms, confusion, and anger (40). Third, it may be culturally unacceptable and legally unenforceable, with many people being cohorted in Fangcang shelter hospitals (41). People fear that they will not receive income and financial compensation during isolation (6). Appropriate communication and awareness need to be enhanced for communities to better accept facility-based isolation. Public acceptance may increase if people are aware of the benefits of institutional isolation and understand that such isolation will result in better protection for their families, associated with better health care, including easier access to practical support.

A comprehensive literature review reveals the advantages and challenges of application of Fangcang shelter hospitals, whereas no literature is available regarding the localization of Fangcang shelter hospital. Also, no research combines the EWM-TOPSIS method with the optimization model to determine the localization.

Thus, the contributions of this study are as follows.

**Contribution 1:** The referral to and triage in Fangcang shelter hospitals are characterized and the localization is discussed. We fill the research gap.
**Contribution 2:** An evaluation framework consisting of the EWM and the TOPSIS is proposed. The framework and variants of the *p*-median model are integrated to determine the localization of Fangcang shelter hospitals.
**Contribution 3:** The time-risk function is introduced; variants of the *p*-median model consider the deterministic and uncertain situations, respectively.
**Contribution 4:** A number of realistic situations such as capacity constraints, deterioration ratios, and service use rates are considered. Thus, the proposed models are generalizable.

In this paper, we first integrate the EWM and the TOPSIS to develop an evaluation framework to identify preliminary selections of Fangcang shelter hospitals; second, we consider the timeliness of isolating and treating patients with different severities of infectious disease. We characterize the referral to and triage in Fangcang shelter hospitals and consider the capacity constraints of Fangcang shelter hospitals and designated higher-level hospitals. The *p*-median problem is a typical class of combinatorial optimization problems, which pertain to the non-deterministic polynomial-time (NP)-hard problems and is more commonly applied in the fields of logistics and facility location (42). Model 1 and Model 2 developed in this paper are variants of the *p*-median model, and both are two-level facility location models with capacity limitations.

## Preliminary Selection Methods

The preliminary location selection of candidate Fangcang shelter hospital is not arbitrary. Fangcang shelter hospitals are distinct from conventional hospitals in location requirements on the grounds of exclusive admission and treatment of patients with infectious diseases. In decision-making of location option, the relevant factors are prerequisites: (1) The risk of pandemic spread and locations in marginal and remote areas far away from densely populated areas and city centers; (2) The number of expected patients to be accommodated in candidate Fangcang shelter hospital locations, and the capacity of each Fangcang shelter hospital to meet the medical needs of a certain number of patients; (3) Optimal location relatively close to the designated hospitals, convenience for the transportation of severely diseased patients and the supply of medical materials.

Entropy weight method is a commonly adopted weighting method to measure the structural complexity. It can characterize the size of information in decision-making (43). TOPSIS is a multicriteria decision-making approach. It sorts the evaluation objects according to their consistency to the ideal solution and evaluates the relative merits among the existing evaluation objects (44). TOPSIS accredits equal weights to each criterion, whereby various criteria would play the different roles during the procedure are ignored (45). In this study, EWM is adopted to calculate the weight of each criterion and reduce the disadvantages of TOPSIS, which adopts equal weights. This method addresses the issue of inaccurate assessment and avoids the single-sided effects of using a single criterion. In summary, the EWM-TOPSIS method has the significant advantages in the comprehensive evaluation of the aspects of actual problems (46), as in selection of candidate Fangcang shelter hospital locations.

### Entropy Weight Method—Calculate the Objective Weight of Each Index

#### Initialize the Original Matrix

Assuming that there are *m* candidate locations and *n* evaluation indexes in the evaluation index system, *y*_*ij*_ is the *j*th index's value in the *i*th candidate location, (*i* = 1, 2,…, *m*; *j* = 1, 2,…, *n*).

(1) $$\begin{array}{r} Y={\left(y_{ij}\right)}_{m\times n}=\left(\begin{array}{cccc} y_{11} & y_{12} & \cdots & y_{1n}\\ y_{21} & y_{22} & \cdots & y_{2n}\\ \vdots & \vdots & \; & \vdots \\ y_{m1} & y_{m2} & \cdots & y_{mn} \end{array}\right) \end{array}$$

#### Standardization of Original Data

The greater the index values in the index system, the better will be the candidate location. These indexes are called positive indexes; other indexes present the opposite trend and are thus called negative indexes.

To ensure that all indexes are positive, convert the negative indexes to positive indexes:

(2) $$\begin{array}{r} x_{ij}={\left(x_{j}\right)}_{max}-y_{ij} \end{array}$$

where *x*_*ij*_ is the index value (*i* = 1, 2, …, *m*; *j* = 1, 2, …, *n*) after conversion.

(3) $$\begin{array}{r} X={\left(x_{ij}\right)}_{m\times n}=\left(\begin{array}{cccc} x_{11} & x_{12} & \cdots & x_{1n}\\ x_{21} & x_{22} & \cdots & x_{2n}\\ \vdots & \vdots & \; & \vdots \\ x_{m1} & x_{m2} & \cdots & x_{mn} \end{array}\right). \end{array}$$

To eliminate the effect of the index dimension and its variation range on evaluation results, standardizing the matrix *X* is necessary to ensure that all the attributes are equivalent and in the same format (non-dimensionalization method).

Then derive the standardized matrix *Z*:

(4) $$\begin{array}{r} Z={\left(z_{ij}\right)}_{m\times n}=\left(\begin{array}{cccc} z_{11} & z_{12} & \cdots & z_{1n}\\ z_{21} & z_{22} & \cdots & z_{2n}\\ \vdots & \vdots & \; & \vdots \\ z_{m1} & z_{m2} & \cdots & z_{mn} \end{array}\right) \end{array}$$

wherein:

(5) $$\begin{array}{r} z_{ij}=\frac{x_{ij}}{\sqrt{\overset{m}{\underset{i=1}{\sum }}x_{ij}^{2}}},\left(i=1,2,\cdots \;,m;j=1,2,\cdots \;,n\right). \end{array}$$

#### Calculation of the Index's Entropy

For the comparative analysis between different indexes, we normalize indexes into numerical values within (0,1) to eliminate the influence of dimensions between indexes. The non-dimensionalized formula is presented as follows:

(6) $$\begin{array}{r} P={\left(p_{ij}\right)}_{m\times n}=\left(\begin{array}{cccc} p_{11} & p_{12} & \cdots & p_{1n}\\ p_{21} & p_{22} & \cdots & p_{2n}\\ \vdots & \vdots & \; & \vdots \\ p_{m1} & p_{m2} & \cdots & p_{mn} \end{array}\right) \end{array}$$

wherein:

(7) $$\begin{array}{r} p_{ij}=\frac{z_{ij}}{\overset{m}{\underset{i=1}{\sum }}}z_{ij},\left(i=1,2,\ldots ,m;j=1,2,\ldots ,n\right),p_{ij}\epsilon \left(0,1\right) \end{array}$$

Derive the entropy value of the *j*th index by (8).

(8) $$\begin{array}{r} e_{j}=-\frac{1}{\ln\;m}\overset{m}{\underset{i=1}{\sum }}p_{ij}\ln\;p_{ij},\text{(}j=1,2,\cdots n). \end{array}$$

#### Calculation of the Index's Entropy Weight

Derive the entropy weight of the *j*th index by (9).

(9) $$\begin{array}{r} \omega_{j}=\frac{1-e_{j}}{\overset{n}{\underset{j=1}{\sum }}\left(1-e_{j}\right)},\overset{n}{\underset{j=1}{\sum }}\omega_{j}=1,\text{(}j=1,2,\cdots n). \end{array}$$

In information theory, the entropy weight represents the useful information value of the evaluation index. The bigger the entropy weight of the index is, the more useful information of the index is. It is the same in reverse.

### Technique for Order Preference by Similarity to Ideal Solution

#### Determination of the Weighted Decision Matrix

Multiplying the columns of the standardized matrix by the corresponding weights yields the weighted matrix, which can be expressed as

(10) $$\begin{array}{r} V=\left[v\right]=\left(\omega_{j}z_{ij}\right)=\left(\begin{array}{cccc} \omega_{1}z_{11} & \omega_{2}z_{12} & \cdots & \omega_{3}z_{1n}\\ \omega_{1}z_{21} & \omega_{2}z_{22} & \cdots & \omega_{3}z_{2n}\\ \vdots & \vdots & \; & \vdots \\ \omega_{1}z_{m1} & \omega_{2}z_{m2} & \cdots & \omega_{3}z_{mn} \end{array}\right) \end{array}$$

ω_*j*_ is the weight of the *j*th index and

(11) $$\begin{array}{r} v_{ij}=\omega_{j}z_{ij},\left(i=1,2,\cdots \;,m;j=1,2,\cdots \;,n\right) \end{array}$$

*v*_*ij*_ are the weighted normalized values.

#### Determination of the Ideal Solution

The ideal solution comprises the optimal value of every attribute from the weighted decision matrix, as shown by (11), and the negative ideal solution comprises the worst value of every attribute from the weighted decision matrix, as shown by (12).


(12) $$\begin{array}{l} \begin{array}{l} V^{+}=\left(v_{1}^{+},v_{2}^{+},\cdots \;,v_{n}^{+}\right)\\ \;=(max\left\{v_{11},v_{21},\cdots \;,v_{m1}\right\},max\left\{v_{12},v_{22},\cdots v_{m2}\right\},\cdots \;,\\ \;max\left\{v_{1n},v_{2n},\cdots \;,v_{mn}\right\}) \end{array} \end{array}$$

(13) $$\begin{array}{l} \begin{array}{l} V^{-}=\left(v_{1}^{-},v_{2}^{-},\cdots \;,v_{n}^{-}\right)\\ \;=(min\left\{v_{11},v_{21},\cdots \;,v_{m1}\right\},min\left\{v_{12},v_{22},\cdots \;,v_{m2}\right\},\cdots \;,\\ \;min\left\{v_{1n},v_{2n},\cdots \;,v_{mn}\right\}). \end{array} \end{array}$$

#### Calculation of the Distances Between Solutions

The distances of every feasible solution from the ideal and negative solutions are calculated by (15) and (16), respectively.


(14) $$\begin{array}{r} D_{i}^{+}=\sqrt{\overset{n}{\underset{j=1}{\sum }}{\left(v_{j}^{+}-v_{ij}\right)}^{2}},\left(i=1,2,\cdots \;,m;j=1,2,\cdots \;,n\right) \end{array}$$

(15) $$\begin{array}{r} D_{i}^{-}=\sqrt{\overset{n}{\underset{j=1}{\sum }}{\left(v_{j}^{-}-v_{ij}\right)}^{2}},\left(i=1,2,\cdots \;,m;j=1,2,\cdots \;,n\right). \end{array}$$

#### Calculation of the Relative Degree of Approximation

The relative degree of approximation is determined by (15).

(16) $$\begin{array}{r} S_{i}=\frac{D_{i}^{-}}{D_{i}^{+}+D_{i}^{-}},\left(0\leq S_{i}\leq ;i=1,2,\ldots ,m\right) \end{array}$$

The candidate locations are ranked according to the value of the relative degree of approximation. The bigger the value, the better is the candidate location. By comparing the numerical values of *S*_*i*_, the set *J* of candidate Fangcang shelter hospital locations after preliminary selection is determined.

## Problem Description and Formulations

**Figure 1:**
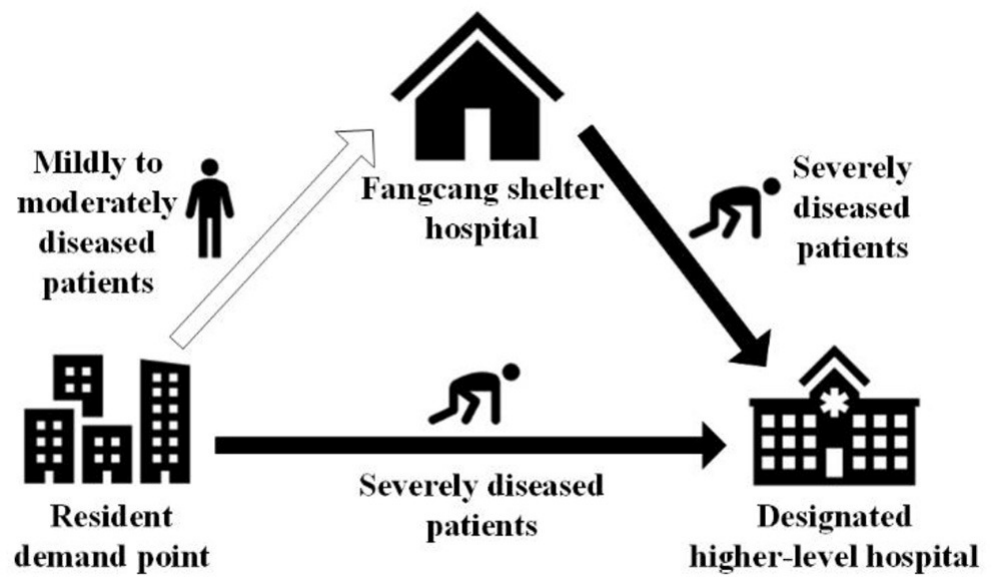
Referral to and triage in a Fangcang shelter hospital.

In the event of pandemic outbreaks, the top priority should be prompt isolation of the infected individuals and protection of the broad masses. The longer the delay, the greater is the risk, and the more elusive is the pandemic control. In China, Fangcang shelter hospital provides a good example in this respect. Figure 1 shows the referral to and triage in a Fangcang shelter hospital.

After a pandemic outbreak, it is essential to promptly transfer mild-to-moderate patients to Fangcang shelter hospitals for isolation and medical care and severe patients to designated higher-level hospitals for professional medical treatment and recovery to avoid the continuous spread of the infectious agents and thus effectively control the pandemic. The proposed models consider the following realistic and essential situations: A portion of the mildly-to-moderately diseased patients in Fangcang shelter hospitals deteriorated, and they were transferred to designated higher-level hospitals. In addition to this, without loss of generality, the mathematical models are based on the following assumptions:

1. Resident demand nodes are represented by the set *I* of discrete variable population distribution nodes, and each street is used as a resident demand node.
2. The set of optimal locations exists in the set *J* of candidate Fangcang shelter hospital locations after preliminary selection.
3. Designated higher-level hospitals only receive and treat severely diseased patients.
4. Each candidate Fangcang shelter hospital location and each designated higher-level hospital has the maximum capacity.
5. The transfer, treatment and triage of patients within cities are only considered.

### The Mathematical Model 1

Model 1 is a variant of the *p*-median model. We introduce in this section the notation used throughout the article and present the objective functions and the constraints.

*Sets*

**Table:** 

*I*	Set of resident demand nodes (*i*ϵ*I, i* = 1,2,…,*m*)
*J*	Set of candidate Fangcang shelter hospital locations after preliminary selection (*j*ϵ*J, j* = 1,2,…,*n*)
*K*	et of designated higher-level hospitals (*k*ϵ*K, k* = 1,2,…,*g*)

*Parameters*

**Table:** 

*w*_*i*_	Number of people at resident demand node *i*
*a*_*i*_	Proportion of diseased patients at resident demand node *i*
*b*_*i*_	Proportion of severely diseased patients among all patients at resident demand node *i* at the onset of the pandemic
*c*_*j*_	Proportion of patients with mild-to-moderate disease deteriorating to severe disease at candidate Fangcang shelter hospital location *j*
*t*_*ij*_	Average travel duration of resident demand node *i* to reach candidate Fangcang shelter hospital location *j*
*t*_*jk*_	Average travel duration of candidate Fangcang shelter hospital location *j* to reach designated higher-level hospital *k*
*t*_*ik*_	Average travel duration of resident demand node *i* to reach designated higher-level hospital *k*
*Q*	Number of Fangcang shelter hospitals to be established
*G*_*j*_	Capacity of candidate Fangcang shelter hospital location *j*
*L*_*k*_	Capacity of designated higher-level hospital *k*
*e*	Number of resident demand nodes served by at least one of the Fangcang shelter hospitals

*Decision Variables*

**Table:** 

*S*_*j*_ϵ{1, 0}	1, if Fangcang shelter hospital is established at candidate Fangcang shelter hospital location *j*
	0, otherwise
*Z*_*ik*_ϵ{1, 0}	1, if patients with mild-to-moderate disease in resident demand node *i* are allocated to designated higher-level hospital *k*
	0, otherwise
*X*_*ij*_ϵ{1, 0}	1, if patients with mild-to-moderate disease in resident demand node *i* are allocated to candidate Fangcang shelter hospital location *j*
	0, otherwise
*Y*_*jk*_ϵ{1, 0}	1, if deteriorated severely diseased patients in candidate location *j* are allocated to designated higher-level hospital *k*
	0, otherwise

Model 1 is developed as follows.

(17) $$\begin{array}{r} \begin{array}{c} min\;Z=\underset{i\in I}{\sum }\underset{k\in K}{\sum }w_{i}a_{i}b_{i}t_{ik}Z_{ik}+\underset{i\in I}{\sum }\underset{j\in J}{\sum }w_{i}a_{i}\left(1-b_{i}\right)t_{ij}X_{ij}\\ +\underset{i\in I}{\sum }\underset{j\in J}{\sum }\underset{k\in K}{\sum }w_{i}a_{i}\left(1-b_{i}\right)c_{j}t_{jk}X_{ij}Y_{jk} \end{array} \end{array}$$

(18) $$\begin{array}{r} s.\;t.\;\underset{j\in J}{\sum }H_{j}=Q \end{array}$$

(19) $$\begin{array}{r} \underset{j\in J}{\sum }X_{ij}=1,\forall i\in I \end{array}$$

(20) $$\begin{array}{r} \underset{k\in K}{\sum }Z_{ik}=1,\forall i\in I \end{array}$$

(21) $$\begin{array}{r} X_{ij}\leq H_{j},\forall i\in I,j\in J \end{array}$$

(22) $$\begin{array}{r} \underset{i\in I}{\sum }X_{ij}\geq eH_{j},\forall j\in J \end{array}$$

(23) $$\begin{array}{r} Y_{jk}\leq H_{j},\forall j\in J,k\in K \end{array}$$

(24) $$\begin{array}{r} \underset{k\in K}{\sum }Y_{jk}=H_{j},\forall j\in J \end{array}$$

(25) $$\begin{array}{r} \underset{i\in I}{\sum }w_{i}a_{i}\left(1-b_{i}\right)X_{ij}\leq G_{j},\forall j\in J \end{array}$$

(26) $$\begin{array}{r} \underset{i\in I}{\sum }w_{i}a_{i}b_{i}Z_{ik}+\underset{i\in I}{\sum }\underset{j\in J}{\sum }w_{i}a_{i}\left(1-b_{i}\right)c_{j}X_{ij}Y_{jk}\leq L_{k},\forall k\in K \end{array}$$

(27) $$\begin{array}{r} H_{j}\in \left\{0,1\right\},\forall j\in J \end{array}$$

(28) $$\begin{array}{r} Z_{ik}\in \left\{0,1\right\},\forall i\in I,k\in K \end{array}$$

(29) $$\begin{array}{r} X_{ij}\in \left\{0,1\right\},\forall i\in I,j\in J \end{array}$$

(30) $$\begin{array}{r} Y_{jk}\in \left\{0,1\right\},\forall j\in J,k\in K \end{array}$$

(31) $$\begin{array}{r} w_{i},Q,e,G_{j},L_{k}\in Z^{+},\forall i\in I,j\in J. \end{array}$$

Objective function (18) minimizes the total population-weighted travel duration of severely diseased patients from resident demand nodes to designated higher-level hospitals, the total population-weighted travel duration of mildly-to-moderately diseased patients from resident demand nodes to Fangcang shelter hospitals, and the total population-weighted travel duration of deteriorated severely diseased patients from Fangcang shelter hospitals to designated higher-level hospitals. Constraint (17) represents that the number of Fangcang shelter hospitals to be established is *Q*; Constraint (18) ensures that patients with mild-to-moderate disease in the resident demand node *i* are assigned to only one Fangcang shelter hospital; Constraint (19) ensures that at the beginning of the pandemic, the severely diseased patients in the resident demand node *i* are assigned to only one designated higher-level hospital; Constraint (20) indicates that candidate location *j* treat patients with mild-to-moderate disease only when a Fangcang shelter hospital is built at a candidate location *j*; Constraint (21) indicates that each Fangcang shelter hospital needs to serve at least *e* resident demand nodes to ensure that the load degree of each Fangcang shelter hospital is relatively balanced, thus avoiding resource waste and reducing the probability of pandemic spread caused by secondary transfer of patients; Constraint (22) indicates that candidate location *j* transfer severely diseased patients to the designated higher-level hospitals only when a Fangcang shelter hospital is built at a candidate location *j*; Constraint (23) ensures that deteriorated severely diseased patients in each Fangcang shelter hospital are assigned to only one designated higher-level hospital; and Constraint (24) indicates the capacity constraints of the candidate Fangcang shelter hospital locations, which ensures that the total of patients with mild-to-moderate disease assigned to the candidate location *j* is less than or equal to the capacity of candidate location *j*. Constraint (25) indicates the capacity constraints of the designated higher-level hospitals, ensuring that the total of patients with severe disease assigned to the designated higher-level hospital *k* is less than or equal to the capacity of the designated higher-level hospital *k*. Constraints (26–29) are 0–1 decision variables. Constraint (30) indicates that some variables are positive integers.

### The Mathematical Model 2

In Model 1, the average travel duration between any two places is a deterministic value. We consider the uncertainty of travel duration and develop Model 2, which is also a variant of the *p*-median model.

According to Chen et al. (47), the time-risk function from demand node *i* to candidate Fangcang shelter hospital location *j* is expressed as:

(32) $$\begin{array}{r} f_{ij}\left(T\right)=\{\begin{array}{cc} \;1 & \;T<l_{ij}\\ \frac{u_{ij}-T}{u_{ij}-l_{ij}} & \;l_{ij}\leq T\leq u_{ij}\\ \;0 & \;T>u_{ij} \end{array}. \end{array}$$

In this function, the travel duration of resident demand node *i* to candidate Fangcang shelter hospital location *j* is uncertain, and the duration value is in the interval [*l*_*ij*_ –*u*_*ij*_].

Supposing the time limit of resident demand node *i* to reach the candidate Fangcang shelter hospital location *j* is *T*. When *T* < *l*_*ij*_, reaching the candidate Fangcang shelter hospital location *j* from the resident demand node *i* in *T* is impossible; therefore, the risk value is equal to 1. When *T* > *u*_*ij*_, the candidate Fangcang shelter hospital location *j* can be reached from the resident demand node *i* in *T*; therefore, the time-risk value is equal to 0. When *l*_*ij*_ ≤ *T* ≤ *u*_*ij*_, a linear function is adopted to represent the corresponding risk value.

Similarly, the time-risk function for the resident demand node *i* to the designated higher-level hospital *k* is

(33) $$\begin{array}{l} f_{ik}\left(T\right)=\{\begin{array}{cc} \;1\; & \;T<l_{ik}\\ \frac{u_{ik}-T}{u_{ik}-l_{ik}}\; & \;l_{ik}\leq T\leq u_{ik}\\ \;0\; & \;T>u_{ik} \end{array} \end{array}$$

The time-risk function from the candidate Fangcang shelter hospital location *j* to the designated higher-level hospital *k* is

(34) $$\begin{array}{r} f_{jk}\left(T\right)=\{\begin{array}{cc} \;1\; & \;T<l_{jk}\\ \frac{u_{jk}-T}{u_{jk}-l_{jk}}\; & \;l_{jk}\leq T\leq u_{jk}\\ \;0\; & \;T>u_{jk} \end{array} \end{array}$$

Model 2 reads as follows, with the same Constraints (17–30).

(35) $$\begin{array}{r} \begin{array}{c} min\;Z=\underset{i\in I}{\sum }\underset{k\in K}{\sum }w_{i}a_{i}b_{i}f_{ik}\left(T\right)Z_{ik}+\underset{i\in I}{\sum }\underset{j\in J}{\sum }w_{i}a_{i}\left(1-b_{i}\right)f_{ij}\left(T\right)X_{ij}\\ +\underset{i\in I}{\sum }\underset{j\in J}{\sum }\underset{k\in K}{\sum }w_{i}a_{i}\left(1-b_{i}\right)c_{j}f_{jk}\left(T\right)X_{ij}Y_{jk} \end{array} \end{array}$$

## Case Study and Results

Xuzhou, Jiangsu Province, China served as an example. As per the administrative distribution of Xuzhou, a total of 30 resident demand nodes are identified for all streets, with 15 candidate Fangcang shelter hospital locations, and 4 designated higher-level hospitals for the treatment of infectious disease.

### Data Resource

The population numbers *w*_*i*_ of each community are derived from the data of Xuzhou Statistical Yearbook 2020, and the population of each demand node was used as weights. In this work, relevant actual data at the time of the pandemic outbreak in Wuhan were used, such as the floor area of Wuhan Gymnasium and its capacity. The capacity *G*_*j*_ of Xuzhou candidate Fangcang shelter hospital locations was estimated based on the floor area of Wuhan Gymnasium in the following way. The capacity of the candidate Fangcang shelter hospital location *G*_*j*_ = (number of beds in Wuhan Gymnasium)/(Wuhan Gymnasium footprint) × (the footprint of candidate location *j*). The identification of designated higher-level hospitals and their capacities *L*_*k*_ were derived from the official websites of these hospitals.

Proportion of the total sick population *a*_*i*_, proportion of severe disease among all patients *b*_*i*_ and proportion of mildly-to-moderately diseased patients deteriorating to severely diseased in Fangcang shelter hospitals *c*_*j*_ were based on actual data of Wuhan during COVID-19 pandemic.

Different travel durations were calculated according to the distance between any two places in the time-risk function, and the upper and lower bound values are derived. Finally, the uncertainty time value interval [*l*_*ij*_ – *u*_*ij*_] between any two places is derived. Assuming that the response time *T* for isolation and rescue is 15 min.

The data of resident demand nodes, candidate Fangcang shelter hospital locations, and designated higher-level hospitals are given in Tables 1–3.

**Table 1:** Coordinates and number of residents at resident demand node *i*.

***i* (No.)**	**Longitude and latitude**	***w_***i***_***
1	117.188372, 34.26608	60,369
2	117.180028, 34.26411	81,182
3	117.153905, 34.25716	47,615
4	117.161256, 34.275161	42,255
5	117.200323, 34.211727	47,097
6	117.200783, 34.244536	27,607
7	117.179484, 34.278059	93,268
8	117.196268, 34.228498	41,131
9	117.177267, 34.224044	24,124
10	117.130913, 34.272531	30,211
11	117.128022, 34.308246	20,072
12	117.105179, 34.308921	13,935
13	117.129148, 34.325718	30,406
14	117.221154, 34.234951	5,913
15	117.232695, 34.28765	65,638
16	117.200181, 34.287339	57,958
17	117.195562, 34.276154	39,318
18	117.195505, 34.305821	40,732
19	117.207516, 34.314247	30,745
20	117.166457, 34.281222	40,378
21	117.142245, 34.309043	36,072
22	117.25, 34.29837	27,203
23	117.270469, 34.294403	37,217
24	117.193946, 34.253947	68,100
25	117.213347, 34.265892	64,295
26	117.247715, 34.26886	51,633
27	117.238175, 34.247926	48,287
28	117.259698, 34.246405	13,887
29	117.256468, 34.246659	14,320
30	117.276251, 34.184719	95,669

**Table 2:** Coordinates and capacity of candidate Fangcang shelter location *j*.

***j* (No.)**	**Longitude and latitude**	***G_***j***_***
1	117.301475, 34.231538	11,089
2	117.23851, 34.227624	455
3	117.197462, 34.253914	2,753
4	117.306177, 34.202956	21,749
5	117.263607, 34.266058	14,077
6	117.256313, 34.269682	1,589
7	117.16915, 34.253934	1,565
8	117.207673, 34.226368	24,322
9	117.182764, 34.209231	16,892
10	117.187335, 34.202369	8,603
11	117.20742, 34.23434	10,609
12	117.221406, 34.218963	9,385
13	117.138684, 34.267088	9,588
14	117.150151, 34.305683	17,205
15	117.258147, 34.297825	6,691

**Table 3:** Coordinates and capacity of designated higher-level hospital *k*.

***k* (No.)**	**Longitude and latitude**	***L_***k***_***
1	117.24619, 34.275971	360
2	117.184448, 34.268087	4,500
3	117.17254, 34.25994	1,800
4	117.202733, 34.27866	1,100

### Computational Results of the Evaluation Framework

The original matrix of indexes for candidate locations for Fangcang shelter hospitals comprises the following factors: risk of spread of the pandemic, number of patients to be accommodated, and accessibility of transport for the transfer of severely diseased patients. The risk of pandemic transmission is determined by the distance between the candidate location and the city center. To reduce the probability of transmission of infectious disease, preference should be given to the urban fringe and remote areas, away from the city center and densely populated areas.

The ease of transport for the transfer of severely diseased patients is determined by the shortest distance from the candidate Fangcang shelter hospital location to each of the designated higher-level hospitals.

The computational results are as follows.

$$\begin{array}{r} Y=\left(\begin{array}{ccc} 12.0449 & 11089 & 6.5511\\ 5.3306 & 455 & 2.6048\\ 0.856 & 2753 & 1.3876\\ 12.8649 & 21749 & 7.6378\\ 7.6789 & 14077 & 2.0014\\ 6.8658 & 1589 & 1.1703\\ 2.9351 & 1565 & 0.4849\\ 2.6335 & 24322 & 2.7145\\ 3.3325 & 16892 & 2.8152\\ 3.5056 & 8603 & 3.354\\ 2.2892 & 10609 & 2.3125\\ 3.9387 & 9385 & 3.6777\\ 6.2151 & 9588 & 3.7821\\ 5.2691 & 17205 & 3.4043\\ 7.2149 & 6691 & 1.7338 \end{array}\right)X=\left(\begin{array}{ccc} 12.0449 & 11089 & 1.0867\\ 5.3306 & 455 & 5.033\\ 0.856 & 2753 & 6.2502\\ 12.8649 & 21749 & 0\\ 7.6789 & 14077 & 5.6364\\ 6.8658 & 1589 & 6.4675\\ 2.9351 & 1565 & 7.1529\\ 2.6335 & 24322 & 4.9233\\ 3.3325 & 16892 & 4.8226\\ 3.5056 & 8603 & 4.2838\\ 2.2892 & 10609 & 5.3253\\ 3.9387 & 9385 & 3.9601\\ 6.2151 & 9588 & 3.8557\\ 5.2691 & 17205 & 4.2335\\ 7.2149 & 6691 & 5.904 \end{array}\right)\\ Z=\left(\begin{array}{ccc} 0.4822 & 0.2268 & 0.0567\\ 0.2134 & 0.0093 & 0.2624\\ 0.0343 & 0.0563 & 0.3259\\ 0.515 & 0.4448 & 0\\ 0.3074 & 0.2879 & 0.2939\\ 0.2749 & 0.0325 & 0.3372\\ 0.1175 & 0.032 & 0.3729\\ 0.1054 & 0.4974 & 0.2567\\ 0.1334 & 0.3454 & 0.2514\\ 0.1403 & 0.1759 & 0.2233\\ 0.0916 & 0.217 & 0.2776\\ 0.1577 & 0.1919 & 0.2065\\ 0.2488 & 0.1961 & 0.201\\ 0.2109 & 0.3518 & 0.2207\\ 0.2888 & 0.1368 & 0.3078 \end{array}\right)P=\left(\begin{array}{ccc} 0.1452 & 0.0708 & 0.0158\\ 0.0642 & 0.0029 & 0.073\\ 0.0103 & 0.0176 & 0.0907\\ 0.155 & 0.1389 & 0\\ 0.0925 & 0.0899 & 0.0818\\ 0.0827 & 0.0101 & 0.0938\\ 0.0354 & 0.01 & 0.1038\\ 0.0317 & 0.1553 & 0.0714\\ 0.0402 & 0.1079 & 0.07\\ 0.0422 & 0.0549 & 0.0621\\ 0.0276 & 0.0678 & 0.0773\\ 0.0475 & 0.0599 & 0.0574\\ 0.0749 & 0.0612 & 0.0559\\ 0.0635 & 0.1099 & 0.0614\\ 0.087 & 0.0427 & 0.0856 \end{array}\right)\\ V=\left(\begin{array}{ccc} 0.1508 & 0.1068 & 0.0123\\ 0.0667 & 0.0044 & 0.0568\\ 0.0107 & 0.0265 & 0.0705\\ 0.161 & 0.2095 & 0\\ 0.0961 & 0.1356 & 0.0636\\ 0.086 & 0.0153 & 0.073\\ 0.0367 & 0.0151 & 0.0807\\ 0.033 & 0.2342 & 0.0555\\ 0.0417 & 0.1626 & 0.0544\\ 0.0439 & 0.0828 & 0.0483\\ 0.0286 & 0.1022 & 0.06\\ 0.0493 & 0.0904 & 0.0447\\ 0.0778 & 0.0923 & 0.0435\\ 0.0659 & 0.1657 & 0.0478\\ 0.0903 & 0.0644 & 0.0666 \end{array}\right) \end{array}$$

With the EWM, the objective weights of the indexes risk of spread of the pandemic, number of patients that can be accommodated, and accessibility of transport for the transfer of severely diseased patients are 0.3127, 0.4709, and 0.2164, respectively.

The scores of the 15 candidate locations (Table 2) were 0.5519, 0.2944, 0.2766, 0.694, 0.598, 0.3743, 0.3166, 0.6032, 0.52, 0.3465, 0.3874, 0.365, 0.4269, 0.5675, and 0.4405. Based on these scores, the eight candidate Fangcang shelter hospital locations with relatively higher scores are Nos.1, 4, 5, 8, 9, 13, 14, and 15.

**Figure 2:**
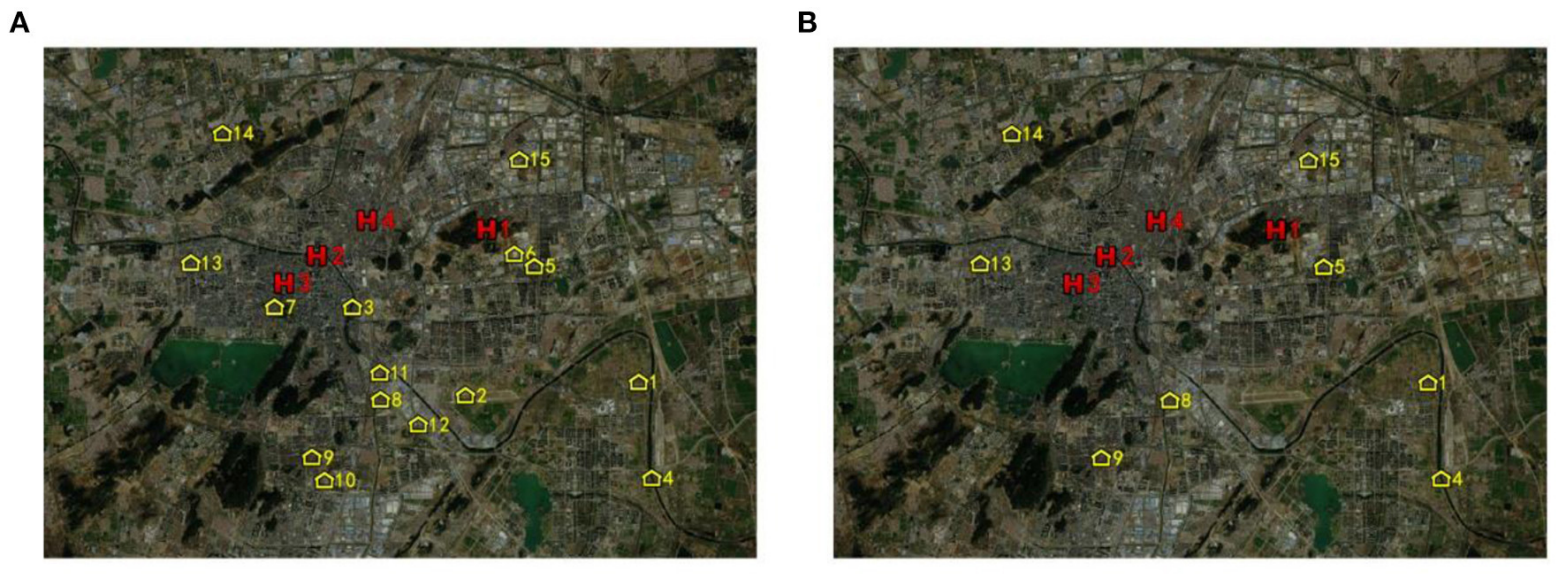
Candidate Fangcang shelter hospital locations **(A)** before preliminary selection **(B)** after preliminary selection.

Figure 2A shows the geographic locations of all candidate Fangcang shelter hospitals before preliminary selection and all designated higher-level hospitals. Figure 2B shows the geographic locations of the eight candidate Fangcang shelter hospital locations after preliminary selection and all designated higher-level hospitals. The yellow house symbols represent candidate Fangcang shelter hospital locations, and the red H symbols represent designated higher-level hospitals.

### Computational Results of Mathematical Model 1

**Figure 3:**
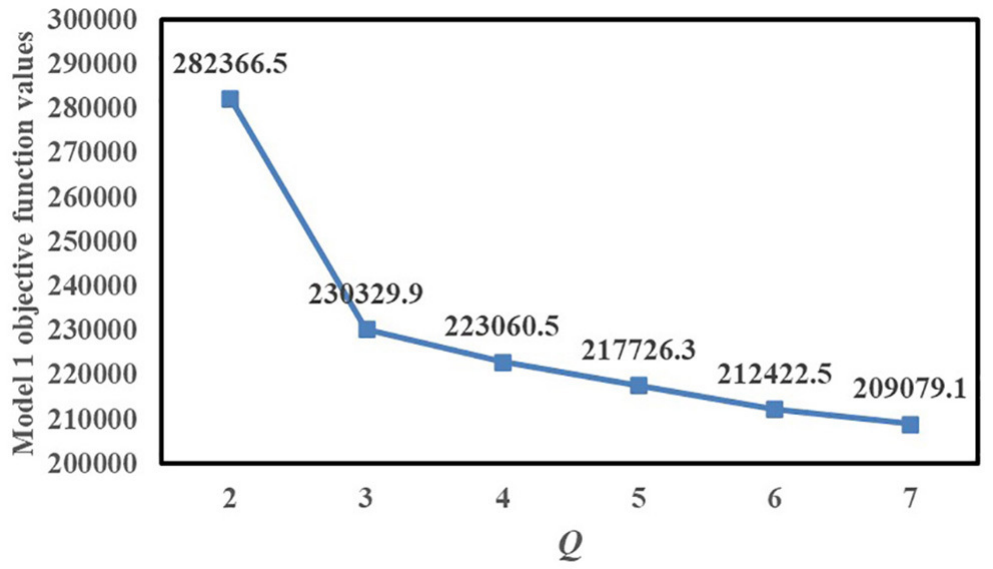
Variation of Model 1 objective function value with *Q*.

*Q* is the number of Fangcang shelter hospitals to be established. Figure 3 shows the variation of the objective function value of Model 1 with *Q*. *Q* considerably affects the value of the Model 1 objective function. As *Q* increases, the total population-weighted travel duration gradually decreases, and when *Q* ≥ 3, the decrease of the Model 1 objective function value slows down. When the number of candidates Fangcang shelter hospital locations is 3, the patients' demand for medical care can be satisfied. Therefore, if Model 1 is used, *Q* can be set to 3.

**Figure 4:**
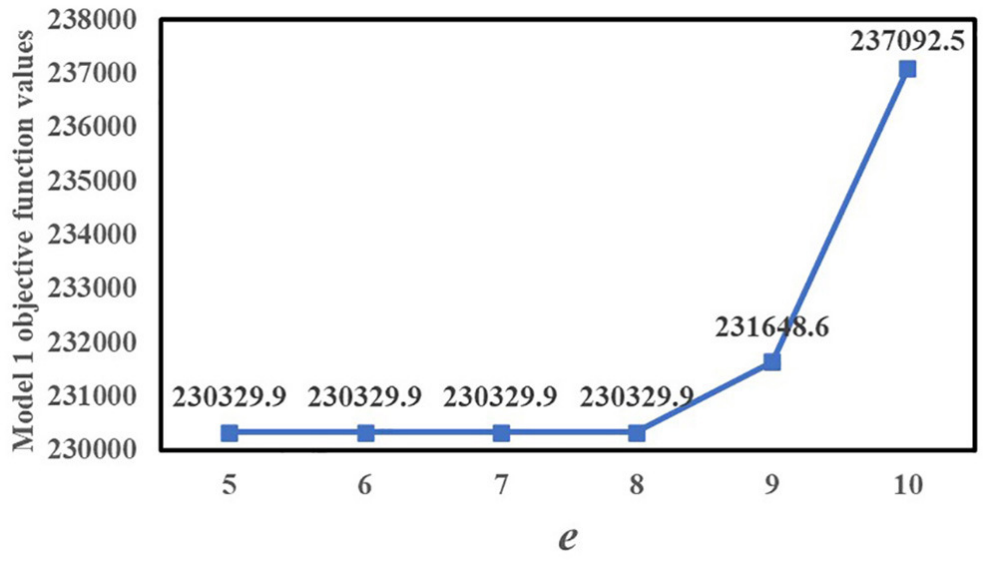
Variation of Model 1 objective function value with *e*.

*e* is the number of resident demand nodes served by at least one of the Fangcang shelter hospitals. Figure 4 shows the variation of the objective function value of Model 1 with *e*. When *e* = 5, 6, 7, and 8, all Model 1 objective function values are 230,329.9, and when *e* > 8, the total population-weighted travel duration gradually increases with *e*. Therefore, if Model 1 is used, *e* can be set to 8, which can ensure the relative balance of load levels for each Fangcang shelter hospital and minimize the total objective function value.

Tables 4, 5 show the computational results according to Model 1, showing that candidate locations No.5, No.8, and No.14 are selected for Fangcang shelter hospitals. Because the capacity constraints of the Fangcang shelter hospitals are considered and the capacity of candidate location No.8 is large, patients with mild-to-moderate disease from 13 resident demand nodes are allocated to candidate location No.8. Meanwhile, the capacity of candidate location No.5 is relatively small; therefore, patients with mild-to-moderate disease from eight resident demand nodes are allocated to candidate location No.5.

**Table 4:** Location and allocation results according to Model 1.

***i* (No.)**	***j* (No.)**	***k* (No.)**
15, 22, 23, 26, 27, 28, 29, 30	5	4
1, 2, 5, 6, 8, 9, 14, 16, 17, 18, 19, 24, 25	8	2
3, 4, 7, 10, 11, 12, 13, 20, 21	14	3

**Table 5:** Allocation results according to Model 1.

***i* (No.)**	***k* (No.)**
23, 26, 28, 29	1
1, 2, 5, 6, 7, 8, 14, 16, 17, 18, 19, 24, 30	2
3, 4, 9, 10, 11, 12, 13, 20, 21	3
15, 22, 25, 27	4

Moreover, because the capacity constraints of the designated higher-level hospitals are considered, and the capacity of designated higher-level hospital No.1 was relatively small, designated higher-level hospital No.1 could not treat severely diseased patients from Fangcang shelter hospitals. It could only treat severely diseased patients from the No.23, No.36, No.28, and No.29 resident demand nodes.

### Computational Results of Mathematical Model 2

**Figure 5:**
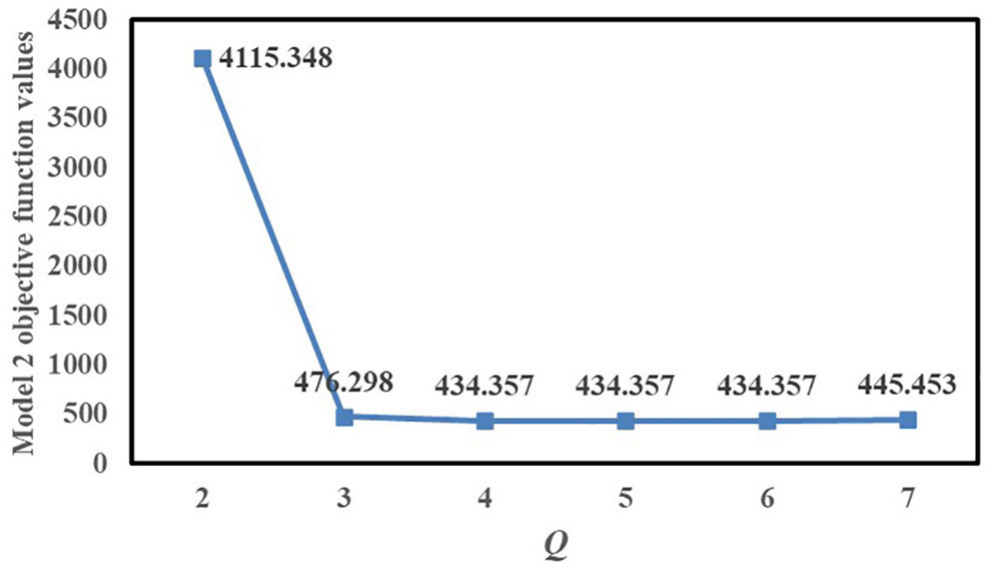
Variation of Model 2 objective function value with *Q*.

*Q* is the number of Fangcang shelter hospitals to be established. Figure 5 shows the variation of the Model 2 objective function value with *Q*. When 2 ≤ *Q* ≤ 6, the total population-weighted time-risk value gradually decreases as *Q* increases, and when 3 ≤ *Q* ≤ 6, the Model 2 objective function value decreases slowly and can meet the patients' demand for medical care so that *Q* can be set to 3.

When *Q* = 4, 5, or 6, the Model 2 objective function values are all 434.357. If all objective function values are 434.357, *Q* is selected to be set to 4 because it can meet the patients' demand for medical treatment and reduce the setup costs of Fangcang shelter hospitals. Furthermore, when *Q* changes from 6 to 7, the objective function value slightly increases.

In summary, if Model 2 is used, *Q* can be set to 3 or 4. Both of these options have advantages and disadvantages, and the decision depends on public health managers' preference and priority for the cost of building Fangcang shelter hospitals and controlling the pandemic; if managers focus more on lower costs, *Q* can be set to 3, and if managers focus more on timely control of the pandemic, *Q* can be set to 4.

**Figure 6:**
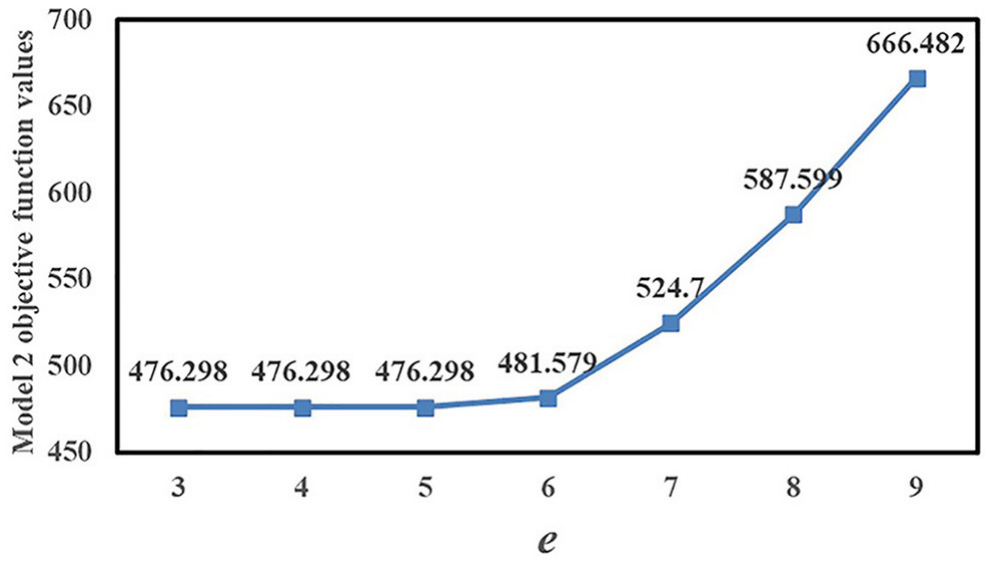
Variation of Model 2 objective function value with *e*.

We now study the scenario of *Q* = 3, and *e* is the number of resident demand nodes served at least by each Fangcang shelter hospital; *e* with the value of the Model 2 objective function is shown in Figure 6. When *e* = 3, 4, or 5, all objective function values are 476.298, and when *e* ≥ 6, the objective function values rise faster; therefore, according to Model 2, *e* can be set to 6. Compared with the scenario of *e* = 7, 8, 9, when *e* =6, the balance of the load degree of each Fangcang shelter hospital is relatively worse. If managers want to make the load degree of each Fangcang shelter hospital more balanced, the time-risk value of pandemic control can be increased to a certain extent. Thus, the location decision depends on managers' tolerance for total population-weighted time-risk value.

Tables 6, 7 show the computational results based on Model 2, in which candidate locations No.5, No.8, and No.13 are selected for the location of Fangcang shelter hospitals. Because the capacity constraints of Fangcang shelter hospitals are considered, and the capacity of candidate location No.8 is larger than those of No.5 and No.13, patients with mild-to-moderate disease from 12 resident demand nodes are allocated to candidate location No.8. In comparison, the capacity of candidate location No.13 is relatively lower; therefore, patients with mild-to-moderate disease from nine resident demand nodes are allocated to candidate location No.13.

**Table 6:** Location and allocation results according to Model 2.

***i* (No.)**	***j* (No.)**	***k* (No.)**
14, 15, 22, 23, 26, 27, 28, 29, 30	5	4
1, 2, 5, 6, 8, 9, 16, 17, 18, 19, 24, 25	8	2
3, 4, 7, 10, 11, 12, 13, 20, 21	13	3

**Table 7:** Allocation results according to Model 2.

***i* (No.)**	***k* (No.)**
28, 30	1
1, 2, 3, 4, 5, 6, 7, 8, 9, 14, 19, 20, 23, 29	2
10, 11, 12, 13, 16, 17, 18, 21, 24, 25	3
15, 22, 26, 27	4

Moreover, because the capacity constraints of designated higher-level hospitals are also considered and the capacity of designated higher-level hospital No.1 is relatively small, designated higher-level hospital No.1 could not treat severely diseased patients from Fangcang shelter hospitals and could only treat severely diseased patients from No.28 and No.30 resident demand nodes.

**Figure 7:**
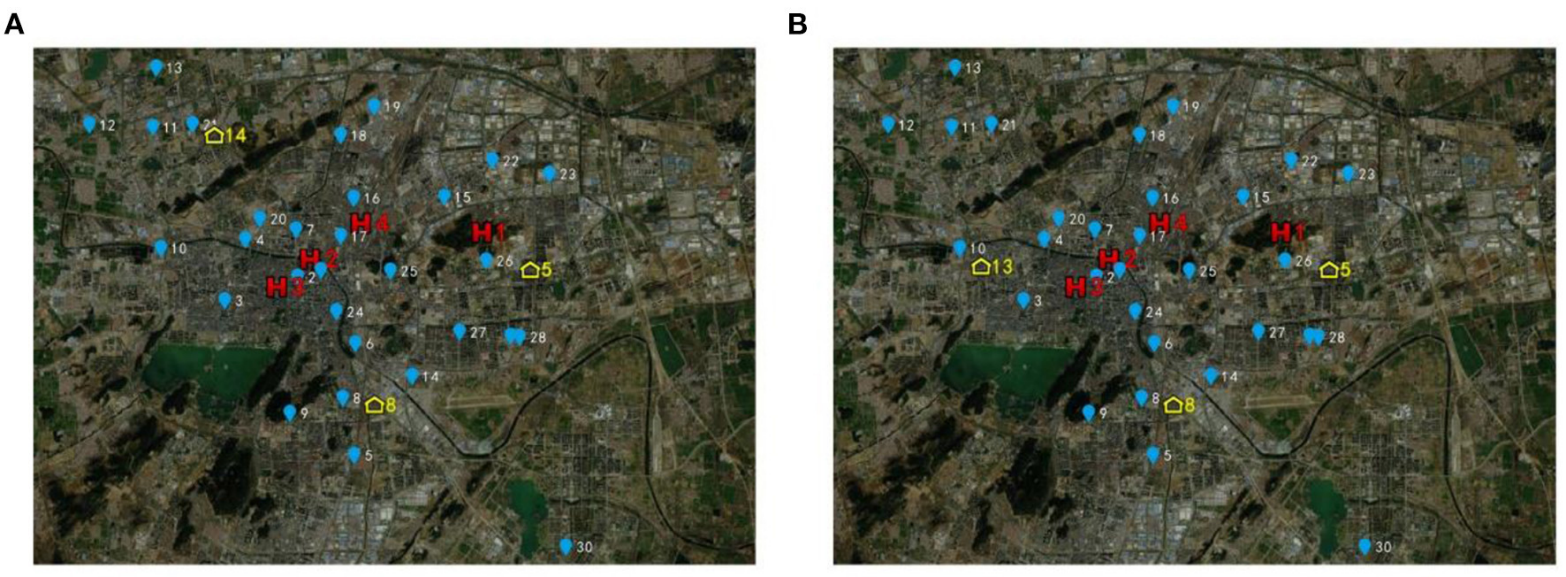
Location results based on the proposed models **(A)** based on model 1 **(B)** based on model 2.

Figure 7A shows the schematic of the location results based on Model 1. Figure 7B shows a schematic of the location results based on Model 2. The blue dots represent resident demand nodes, the yellow house symbols represent identified candidate Fangcang shelter hospital locations, and the red H symbols represent designated higher-level hospitals.

## Discussion

Nos.1, 4, 5, 8, 9, 13, 14, and 15 candidate locations were selected after preliminary selection. Their comprehensive evaluation values were relatively high among all candidate locations. It can be observed from Figure 2 that the candidate locations after preliminary identification are at the outskirts of the city, far from the city center and densely populated areas, which can effectively reduce the spread of the pandemic and cross infection. Meanwhile, these candidate locations had a relatively large capacity of 11,089, 21,749, 14,077, 24,322, 16,892, 9,588, 17,205, and 6,691, respectively, to meet the demand of patients for medical treatment. In addition, these selected candidate locations are relatively adjacent to higher-level designated hospitals than other candidate locations so that it will be more convenient to transfer patients with deterioration and transport medical supplies. The evaluation framework can determine the preliminary selection effectively and efficiently. It also embodies the unique location criteria and requirements of Fangcang shelter hospital which are distinct from conventional hospitals.

The computational results of Model 1 and Model 2 share some similarities. First, both results indicate that each Fangcang shelter hospital is covered by the corresponding higher-level designated hospitals, which can ensure the transfer and treatment of severely diseased patients. All the patients in the resident demand nodes can be treated. It can be seen from Figure 7 that Fangcang Shelter Hospital is evenly distributed in Xuzhou, which can well cover the resident demand nodes in each district and reduce the total travel duration and total travel risk of patients. Second, candidate location No.5, with a capacity of 14,077, is in Yunlong District with a population of ~3.5 million. The candidate location No.8, with a capacity of 24,322, is in Quanshan District with a population of approximately 5.6 million. It is in line with the realistic situation of population and demand in Yunlong District and Quanshan District. In addition, the section of the “Case Study and Results” also verifies that the computational results are consistent with the capacity constraints of Fangcang shelter hospitals and higher-level designated hospitals. The computational results validate that both models are reasonable. Third, as *e* gradually increases, the trend of the objective function values of the two models is similar. As *e* gradually increases, the objective function values are all equal, and when *e* increases to a specific value, the objective function value increases accordingly. Managers can set reasonable values of *e* based on the discussion and suggestions of the “Case Study and Results” section. Fourth, whether using Model 1 or Model 2, the computational results both include candidate locations No.5 and No.8. In other words, the candidate locations No.5 and No.8 are both among the optimal locations, regardless of whether the travel duration is deterministic or uncertain. If an outbreak occurs in Xuzhou like the one that once occurred in Wuhan, the government and public health managers should note that candidate locations No.5 and No.8 cannot be ignored when making localization decisions.

There are also two differences between the models we proposed. The first is the trend of the objective function values with *Q* for Model 1 and Model 2. For Model 1, *Q* considerably affects the value of the objective function. While for Model 2, As *Q* increases, the total population-weighted travel duration gradually decreases. When *Q* ≥ 3, the decrease slows down. For Model 2, when 2 ≤ *Q* ≤ 6, the total population-weighted time-risk gradually decreases as *Q* increases, and when 3 ≤ *Q* ≤ 6, the Model 2 objective function value decreases slowly. When *Q* = 4, 5, or 6, the Model 2 objective function values are all 434.357. Furthermore, when *Q* changes from 6 to 7, the objective function value slightly increases; therefore, according to the computational results of Model 2, a greater number of candidate locations does not necessarily lead to more timely control of the pandemic. But according to the computational results of Model 1, more candidate locations lead to more timely control of the pandemic. Secondly, in identifying the third location, Model 1 selects candidate location No.14, whereas Model 2 selects candidate location No.13.

From analyses of the results of the computational experiments, we identified that Model 1 is superior, with the reason listed as follows. When *Q* = 4, 5, or 6, the Model 2 objective function values are all 434.357. After the time-risk function is introduced, the resident demand node *i* is coarsely classified relative to the candidate Fangcang shelter hospital location *j*. For example, there are several demand nodes closer to a candidate location and their time-risk values relative to that candidate location are all 0, resulting in no difference in the time-risk value. The use of the time-risk function, while capturing the uncertainty of travel time, leads to homogenization of some resident demand nodes relative to the candidate Fangcang shelter hospital location. The differences between the resident demand nodes can't be captured. This may not be realistic. In Model 1, the average travel duration of each resident demand node *i* to reach the candidate Fangcang shelter hospital location *j* are different, all objective function values are different in the case of different *Q*.

In addition, the budget is needed to be considered by managers, because the budget is often limited. Managers usually want to avoid the construction and operation costs of building redundant hospitals. Setting up the right number of hospitals will not only serve the purpose of pandemic control but will also avoid waste. If managers want to prevent and control outbreaks in a timelier manner, there is some additional cost and expense, and if managers want to spend less of budget, there is some additional travel duration and risk. Managers need to weigh preferences and priorities between the cost and objective function values.

In this study, there are three limitations: (i) In the evaluation framework, only three main factors of realistic requirements are considered in the preliminary selection. (ii) The transfer of patients between cities is not considered; the treatment and triage of patients are only considered within cities. (iii) The data in this study are based on the actual data of the Wuhan pandemic, while, the severity of the pandemic situation in different areas is not similar. Firstly, it is understood that many other factors affect the location of Fangcang shelter hospital, such as away from the urban water source, away from key areas related to the urban operation, construction cost, and operation cost. The choice and priority of these factors depend on the actual requirements of pandemic control and the preferences of the government and managers. Secondly, it is possible that cities are not in isolation. On the one hand, people in less developed areas may go to better-developed areas for medical care and treatment. On the other hand, medical staff and resources from other areas may also assist areas with severe pandemic outbreaks. Thirdly, scientific and effective methods are needed to accurately predict the number of infectious disease patients and severely diseased patients. The applicability of the model has yet to be increased.

Future research could focus on considering more location factors, the preliminary selection evaluation framework will be modified, to meet the requirements of pandemic prevention and control more comprehensively. Besides, the number of patients and the number of severely diseased patients can be predicted using infectious disease models such as Susceptible–Exposed–Infectious–Recovered (SEIR), Susceptible–Exposed–Infectious–Quarantined–Recovered (SEIQR), and Susceptible–Exposed–Infectious–Hospitalized–Recovered (SEIHR), and then making optimal location decisions for Fangcang shelter hospitals. Lastly, the location of Fangcang shelter hospitals of different levels, functions, and scales will be further considered in the future.

## Conclusion

The establishment of Fangcang shelter hospitals can effectively relieve the pressure on the healthcare system. This work fills the research gap of the location of Fangcang shelter hospitals. A novel two-stage location method is proposed, including an evaluation framework and variants of the *p*-median model. Policy makers can make decisions quickly based on the two-stage location method proposed in this study.

## Data Availability Statement

The raw data supporting the conclusions of this article will be made available by the authors, without undue reservation.

## Author Contributions

BH conceived and designed the study. WC developed and validated the proposed models. TY and GJ collected the data. All authors contributed to the article and approved the submitted version.

## Funding

This work was supported by the Ministry of Humanities and Social Science Education Project (No. 19YJC630182), Science and Technology Program Project of Xuzhou (No. KC20200), Scientific Research Foundation for Excellent Talents of Xuzhou Medical University (No. D2019004), and Postgraduate Research and Practice Innovation Program of Jiangsu Province (Nos. SJ CX22_1291 and SJCX21_1157).

## Conflict of Interest

The authors declare that the research was conducted in the absence of any commercial or financial relationships that could be construed as a potential conflict of interest.

## Publisher's Note

All claims expressed in this article are solely those of the authors and do not necessarily represent those of their affiliated organizations, or those of the publisher, the editors and the reviewers. Any product that may be evaluated in this article, or claim that may be made by its manufacturer, is not guaranteed or endorsed by the publisher.
